# Supplementary material for: Visual Modulation of Human Responses to Support Surface Translation
Source: Front Hum Neurosci. 2021 Mar 4;15:615200. doi: 10.3389/fnhum.2021.615200 (PMC7969526; doi:10.3389/fnhum.2021.615200)
Supplement: Supplementary file 1 [file Table_1.docx]

**Appendix**

Below (A) the technical version of the DEC model is presented and (B) details of the simulations with the double inverted pendulum (DIP) model (compare Fig. 4 of main text) are given.

**(A) DEC model (technical version)**

The DEC model used for describing the postural responses to support surface translation in the body sagittal plane for the *SIP* scenario is depicted in technical form below in Fig. A1 (for a simplified non-technical version, see Ankle Module in Fig. 3). Its input signal is the desired body in space sway bs! (=0°, for desired vertical body orientation in space). It commands the *Neural Controller* (*PD, proportional-derivative)* after a ‘lumped’ *time delay* to produce a desired voluntary movement or pose of the body COM in space. To this end, the proprioceptive negative feedback (at the bottom) closes the loop of the *Servo Mechanism.* The servo is able to execute the desired movement or pose even in the presence of external disturbances in that its input includes disturbance estimation signals (via Disturbance Compensation Loops on top) with negative sign^[[1]](#footnote-1)^. These signals command the servo to provide the extra ankle torque that is required to compensate for the external disturbances (using the building blocks *Sensory Systems* and *Disturbance Estimators*)*.*

The block Sensory Systems represents the processing and fusion of sensory signals in two sensor fusion boxes:

(A) *Sensor fusion box F1.* It provides the sensory estimate of the body COM lean in space$, \hat{\mathrm{bs}}$, for disturbance compensation of the gravitational ankle torque. It combines

(a) the proprioceptive body-to-foot lean signal *prop* (here preferred over a presumed noisier vestibular body-in-space signal; see Lippi et al. 2020),

(b) the signal vis, which codes the visual body lean angle (perceived with both EO/CI, and EO/SI) and

(c) the visual velocity signal obtained with EO/CI$\dot{\left( \mathrm{vis} \right),}$ after mathematical integration.

(B) *Sensor fusion box F2.* It provides the disturbance estimate of support translational acceleration. It combines

(a) the vestibular heady-in-space linear acceleration signal $\ddot{\left( \mathrm{vest} \right)}, considered here to be small, with$

(b) the above visual velocity signal $\dot{\left( \mathrm{vis} \right)}$of tangential head-to-scene motion, after its mathematical differentiation.

**Fig. A1.** Single inverted pendulum disturbance estimation and compensation model (*SIP DEC model*). The input ('IN') leads to a body lean response ('Out') that is shaped by the Neural Controller, Biomechanics, Sensory Systems, and two Disturbance Estimators. The latter deal with: (i) The gravitational ankle torque evoked by body lean and (ii) the force from body inertia (T_in_) arising with support translational acceleration. Abbreviations: Tin, inertial torque; Tact, active torque; Tpas, passive torque; Tc, control torque; BF, bf, body-foot; BS, body COM in space; HS, head in space; m, mass of body above ankle joints; g, gravitational constant; bs, body-space; vest, vestibular; prop, proprioceptive; vis, visual; wf, weighting factor; th, threshold; F1 and F2, sensor fusions. Further details in the text.

In view of the relatively high number of processing variables in the model versus the small number of testing variables (EC, EO/SI, and EO/CI), we choose a simplifying workaround that would enable us to reproduce in model simulations the experimental data. First, we refrained from making assumptions on specific signal fusion processing in the boxes F1 and F2 (e.g. fusion in some noise-optimal way) but assumed summation of the sensory signals. Furthermore, we assumed for the sake of simplicity a fixed value of zero for the magnitude of the thresholds (*th*) and of unity for the weighting factors (*wf*) in the disturbance estimators. This allowed us to estimate for each of the sensory input signals a gain value across the visual stimulus conditions EC, EO/SI, and EO/CI, and by this, post hoc, to approximately replicate the experimental data in the model simulations. Model implementation and simulations were performed using Simulink® (The Mathworks, Natick, USA). Estimates of body mass and of the height of the mass above the ankle joints of our subjects were derived using the anthropometric tables of Winter (2009).

A finding mentioned in the main text was that not only visual information had a damping effect on the response to the translation stimulus, but also the biomechanics of the sway of the upper body segments in the hip joints. Below we give a formal description of this phenomenon in the form of double inverted pendulum model simulations as well as of a biomechanical description.

***(B) DIP model simulations***

These simulations addressed the EC responses of our subjects and the observation that the angular excursions of subjects’ upper body (HAT, for head-arm-trunk) tended to exceed the excursions of their leg segment, as already shown before in Lippi et al. (2020). This led us to investigate the biomechanical effect of intersegmental coupling between the HAT and the leg segment on the excursion of the COM (which is located slightly above the upper end of the leg segment). In particular, simulating the translation-evoked PRTS sway responses, we compared the effect of a very high passive hip stiffness (to mimic a SIP kind of response behaviour; Fig. 2A, panel A) to that of an intermediate hip stiffness (with DIP like response behaviour, similarly to that observed experimentally; Fig. A2, panel B). Note in the responses of panel B the pronounced reduction in body COM excursion in space (BS curve).

**Fig. A2.** Simulations using the DIP model of Fig. 4 in main text. (A) Within a range of high passive hip stiffness, the translation stimulus produced a SIP-like response behavior where angular excursion of the COM in space (BS), trunk segment in space (TS), and leg segment in space (LS) coincide (note their superposition in gain, phase and coherence FRFs). (B) Repetition of the simulation with reduced hip joint stiffness. Note damping effects, by which the peaks of BS and LS gain become reduced to almost half (and a negative peak in the coherence FRF is extinguished).

As described formally below, the reduction of the COM excursion can be attributed to a biomechanical effect from intersegmental coupling. An explanation for a damping of leg segment and COM excursions by the trunk excursion in the hip joints as shown in Fig. A2, panel b, can be given as follows.

The linearized angular position of the COM is

$BS=\left[ (TS {h_{t}+LS l_{L}) m}_{T}+LS h_{L}m_{L} \right]/(m_{B}h_{B})$,

where $h_{t}$ is the height of the trunk’s COM with respect to the hip joint, $l_{L}$ is the length of the leg (from ankle joint to hip joint), $h_{B}$ the height of the body COM with respect to the ankle joint (approximated as constant, not dependant on the trunk-to-leg angle), while $m_{T}$,$m_{L}$, and $m_{B}$ are the masses of the trunk (HAT), of the leg segment and of the body, respectively. The parameters relative to the whole body are a combination of the ones relative to the body segments: $m_{B}=m_{L}+m_{T}$ and $h_{B}=\left[ ( {h_{t}+l_{L}) m}_{T}+ h_{L}m_{L} \right]/m_{B}$. The center of mass of the upper body is relatively close to the hip joint and hence $h_{B}\tilde{=}l_{L}$ and $BS\tilde{=}LS$. Specifically in the presented simulations $l_{L}=0.8543\mathrm{cm}$ and $h_{B}=0.9296\mathrm{cm}$. This implies that allowing the upper body to independently sway (DIP model) does not affect the total body sway $BS$ directly by a significant degree. On the other hand, the torque produced in the hip joint, mainly by passive stiffness and short-latency proprioceptive stiffness, has a damping effect on the overall system as shown in Fig. 2B-Appendix. More in detail, when the support surface is translated, the effect of upper body inertia on the leg segment is mediated by the hip stiffness. Using again a linearized description, such torque can be expressed as

${\tau_{TL}}_{direct}=\frac{\ddot{X}_{FS}}{s^{2}}\frac{({K_{p}}_{hip}+s{K_{d}}_{hip})}{(1+{K_{p}}_{hip}+s{K_{d}}_{hip})}\frac{{I_{t}h}_{L}m_{L}-{{I_{l}h}_{t}m}_{T}}{I_{l}I_{t}}$,

where ${K_{p}}_{hip}$ is the proportional (stiffness) and ${K_{d}}_{hip}$ is the derivative (damping) coefficient describing the passive reaction of the hip joint, and $I_{l}$and $I_{T}$ are the moment of inertia of the leg and the trunk respectively, computed around the supporting joint. Notice that this expression considers for simplification only the direct effect of the support surface translation and no other indirect effect such as the gravity torque or the active ankle response associated with the induced body sway. The second term on the right side of the equation tends towards unity as the hip stiffness goes to infinity (SIP), and to zero as the stiffness goes to zero. This means that with a compliant DIP model the direct effect of the upper body inertia on body sway is reduced. The third term of the equation is a constant determined by the body mass distribution. This constant is positive with the parameters used in the presented simulations ($\sim4.44 \boldsymbol{m}^{-1}$).

The above suggests that there is an advantage in allowing for hip movements in the disturbance scenario. Although this may be reminiscent of the active hip strategy that tends to become involved in, or used when ankle torque is insufficient (Nashner and McCollum 1985), or with active hip and knee joint involvement in external push compensation, the strategy presented here consists in letting the hip joint be compliant. This represents a relevant factor for the balancing in the here presented stimulus scenario, i.e. support surface translation. Generally, our view is consistent with that of Versteeg et al. (2016) according to whom minimizing COM motion comes with an involvement of the hip, which does not, as our data shows, necessarily require an active hip strategy, since setting a passive hip stiffness in our simulations sufficed to produce the effect.

References

Lippi, V., Assländer, L., Akcay, E., &. Mergner, T. (2020). Body sway responses to pseudorandom support surface translations of vestibular loss subjects resemble those of vestibular able subjects. Neurosci Letters 736

Nashner, L.M., McCollum G. (1985) The Organization of human postural movements: A formal basis and experimental synthesis. Behavioral and Brain Sciences 1: 135-150.

Versteeg, C. S., Ting, L. H., & Allen, J. L. (2016). Hip and ankle responses for reactive balance emerge from varying priorities to reduce effort and kinematic excursion: A simulation study. Journal of biomechanics, 49(14), 3230-3237.

Winter, D. A. (2009). Biomechanics and motor control of human movement. Hoboken, NJ, USA: John Wiley & Sons Inc. (See original data of Dempster, W.T. (1955) The anthropometry of body action. [Ann. New York Acad. Sci.,](https://nyaspubs.onlinelibrary.wiley.com/journal/17496632) [Vol. 63/ 4](https://nyaspubs.onlinelibrary.wiley.com/toc/17496632/1955/63/4)).

1. Their functionality is here, noticeably, equivalent to compensatory feed-forward mechanisms. This type of mechanism is denoted in German textbooks of control theory as „Störgrößen-Aufschaltung“. [↑](#footnote-ref-1)
